# Genetic diversity and genetic structure of the Siberian roe deer (*Capreolus pygargus*) populations from Asia

**DOI:** 10.1186/s12863-015-0244-6

**Published:** 2015-08-18

**Authors:** Yun Sun Lee, Nickolay Markov, Inna Voloshina, Alexander Argunov, Damdingiin Bayarlkhagva, Jang Geun Oh, Yong-Su Park, Mi-Sook Min, Hang Lee, Kyung Seok Kim

**Affiliations:** Conservation Genome Resource Bank for Korean Wildlife, College of Veterinary Medicine, Seoul National University, Gwanak-gu, Seoul 151-742 Republic of Korea; Institute of Plant and Animal Ecology Urals Branch of Russian Academy of Sciences, Yekaterinburg, 620144 Russia; Lazovsky State Nature Reserve, Lazo, Primorsky Krai 692980 Russia; Institute for Biological problems of Cryolihtozone Siberian Branch of Russian Academy of Sciences, Yakutsk, 677980 Russia; Department of Molecular Biology and Genetics, National University of Mongolia, Ulaanbaatar, 210646 Mongolia; Research Institute for Hallasan, Jeju Special Self-Governing Province, Jeju, 690-815 Republic of Korea; Department of Conservation Ecology, National Institute of Ecology, 1210, Geumgang-ro, Maseo-myeon, Seocheon-gun, Chungcheongnam-do 325-813 South Korea; Department of Ecology, Evolution, and Organismal Biology, Iowa State University, Ames, IA 50011 USA

**Keywords:** Microsatellite, Gene flow, Genetic diversity, Genetic structure, Siberian roe deer, *Capreolus pygargus*

## Abstract

**Background:**

The roe deer, *Capreolus* sp., is one of the most widespread meso-mammals of Palearctic distribution, and includes two species, the European roe deer, *C. capreolus* inhabiting mainly Europe, and the Siberian roe deer, *C. pygargus*, distributed throughout continental Asia. Although there are a number of genetic studies concerning European roe deer, the Siberian roe deer has been studied less, and none of these studies use microsatellite markers. Natural processes have led to genetic structuring in wild populations. To understand how these factors have affected genetic structure and connectivity of Siberian roe deer, we investigated variability at 12 microsatellite loci for Siberian roe deer from ten localities in Asia.

**Results:**

Moderate levels of genetic diversity (*H*_E_ = 0.522 to 0.628) were found in all populations except in Jeju Island, South Korea, where the diversity was lowest (*H*_E_ = 0.386). Western populations showed relatively low genetic diversity and higher degrees of genetic differentiation compared with eastern populations (mean *Ar* = 3.54 (east), 2.81 (west), mean *F*_ST_ = 0.122). Bayesian-based clustering analysis revealed the existence of three genetically distinct groups (clusters) for Siberian roe deer, which comprise of the Southeastern group (Mainland Korea, Russian Far East, Trans-Baikal region and Northern part of Mongolia), Northwestern group (Western Siberia and Ural in Russia) and Jeju Island population. Genetic analyses including AMOVA (*F*_RT_ = 0.200), Barrier and PCA also supported genetic differentiation among regions separated primarily by major mountain ridges, suggesting that mountains played a role in the genetic differentiation of Siberian roe deer. On the other hand, genetic evidence also suggests an ongoing migration that may facilitate genetic admixture at the border areas between two groups.

**Conclusions:**

Our results reveal an apparent pattern of genetic differentiation among populations inhabiting Asia, showing moderate levels of genetic diversity with an east-west gradient. The results suggest at least three distinct management units of roe deer in continental Asia, although genetic admixture is evident in some border areas. The insights obtained from this study shed light on management of Siberian roe deer in Asia and may be applied in conservation of local populations of Siberian roe deer.

**Electronic supplementary material:**

The online version of this article (doi:10.1186/s12863-015-0244-6) contains supplementary material, which is available to authorized users.

## Background

The family Cervidae is widely distributed throughout Eurasia and includes 40 species of deer [1]. The roe deer (*Capreolus* Gray, 1821) is one of the most widespread meso-mammals in Cervidae and includes two species, the smaller European roe deer (*C. capreolus* Linnaeus, 1758) and the larger Siberian roe deer (*C. pygargus* Pallas, 1771). The two species of deer are distinguished mainly by differences in morphology and karyotype. The Siberian roe deer is distributed in the Palaearctic throughout continental Asia [2] and some parts of Eastern Europe [3]. Although the classification of subspecies is still controversial, it is widely accepted that the Siberian roe deer comprises of at least three subspecies, *C. pygargus pygargus* (from Volga river to Lake Baikal and Northeastern Russia), *C. pygargus tianschanicus* (or *C. c. bedfordi* Thomas, 1908) (Tianshan mountain, Mongolia, Russian Far East and Korea) and *C. pygargus melanotis* Miller, 1911 (Eastern Tibet, and Gansu and Sichuan Province, China).

For mammal species such as Siberian roe deer, which is distributed across extensive geographical range, contemporary level of genetic variation and population structure may be shaped by interaction of both natural and anthropogenic factors [4, 5]. Especially numerous human activities, such as habitat destruction/fragmentation, hunting, and human-mediated translocation, have influenced distribution, population structure, and genetic diversity of natural wildlife during the last few centuries [6-8]. Fossil records report that Siberian roe deer territory was once connected to the northern Caucasus [9]. However, population size drastically diminished supposedly because of overhunting in Western Siberia and Northeastern Siberia during the 19th and 20th centuries [10]. Regardless, the original historic distribution has almost completely recovered.

Population genetics and phylogeography of European roe deer have been well studied [11–19]. Most studies using mitochondrial and nuclear markers for European roe deer revealed geographic pattern in the population structure, with generally high levels of genetic variation. The Siberian roe deer is relatively less studied and most of the genetic studies of the species have been obtained from phylogenetic inferences using mitochondrial DNA sequence data. These studies using mtDNA demonstrated that Siberian roe deer can be divided into several major clusters with geographic patterns; the cluster in eastern Siberia and the western Siberia [20, 21]. In contrast, some phylogeographic studies have reported no apparent geographic pattern of genetic variation among the broadly sampled Siberian roe deer [19, 22].

Overall, population boundaries and the genetic structuring of the Siberian roe deer remain unclear and the classification of *C. pygargus* subspecies is still under debate. Although phylogenetic studies using mtDNA sequences provided valuable information regarding the genetic relationship and phylogeographic inferences of the Siberian roe deer, studies on population genetics using the fast-evolving nuclear makers, such as microsatellites, can provide additional information to better understand the present status of genetic diversity and population structure of geographic Siberian roe deer in Asia.

In this study, we investigated microsatellite variability for Siberian roe deer collected throughout Asia to examine the level of population genetic structure and the amount of genetic variation of Siberian roe deer. These data were applied to discuss how historical and demographic dynamics have affected the recent and past population genetic structure of Siberian roe deer.

## Results

### Genetic variability of Siberian roe deer

**Fig. 1:**
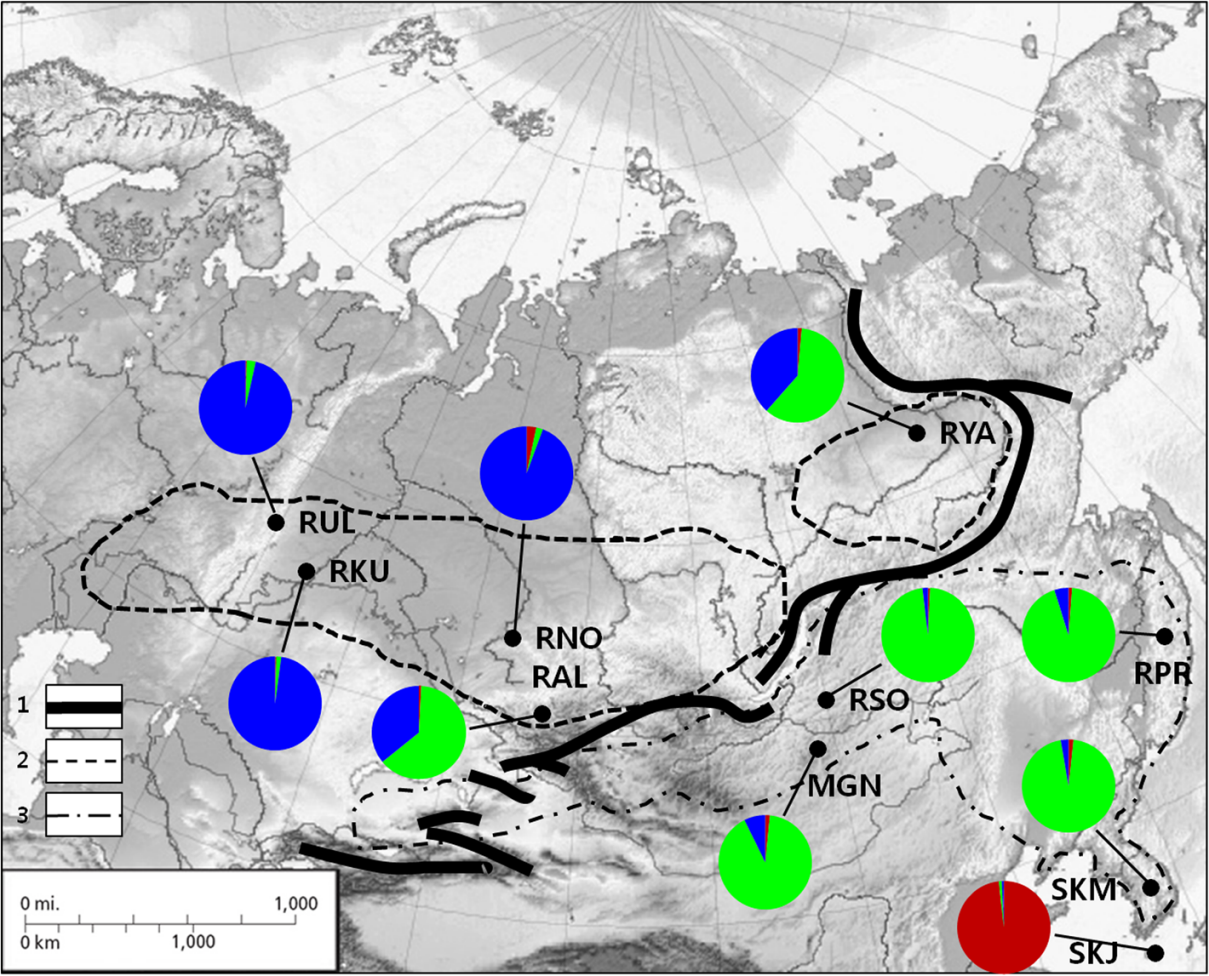
Sampling location and subspecies range of Siberian roe deer, *C. pygargus.* Pie charts of membership proportions of each sampled population inferred by structure analysis (*K* = 3). 1: Main Mountain ranges [2], 2: *C.p.pygargus*, 3: *C.p.tianschanicus*. SKJ: South Korea, Jeju (N = 33), SKM: South Korea Mainland (N = 31), RPR: Russia, Primorsky Krai (N = 30), RYA: Russia, Yakutia (N = 18), RSO: Russia, Sokhondinsky (N = 9), MGN: Mongolia, Northern part (N = 12), RAL: Russia, Altay (N = 5), RNO: Russia, Novosibirsk (N = 7), RUR: Russia, Ural (N = 23), RKU: Russia, Kurgan (N = 21). Base image is created by Uwe Dedering and licensed under the Creative Commons Attribution-Share Alike 3.0 Unported license (CC BY-SA). Fig. 1 is reproduced in this study under the license. https://commons.wikimedia.org/wiki/File:Asia_laea_relief_location_map.jpg

Genetic characteristics of 12 microsatellite loci from Siberian roe deer sampled at each location are shown in Additional file 1: Table S1. Source information and characteristics of 12 microsatellite loci from other species are shown in Additional file 1: Table S2. A total of 122 alleles were detected for 189 individuals of ten Siberian roe deer populations (Fig. 1); Jeju, South Korea (SKJ), Mainland South Korea (SKM), Primorsky Krai, Russia (RPR), Yakutia, Russia (RYA), surroundings of Sokhondinsky Zapovednik (nature reservation), Russia (RSO), Northern part of Mongolia (MGN), Altaisky Krai, Russia (RAL), Novosibirskaya Oblast’, Russia (RNO), Sverdlovskaya oblast’ , Ural, Russia (RUL) and Kurganskaya Oblast’ , Russia (RKU).

The number of alleles per locus varied from 2 (BM25) to 24 (MB757) with a mean of 10.17. Microsatellite loci showed various levels of polymorphism, with the polymorphism information content (PIC) values ranging from 0.062 (IDVGA29) to 0.926 (BM757). Most loci, except IDVGA29, showed moderate to high polymorphism. Private alleles were observed in most populations except Mid-west Siberia (RAL and RNO), but all private alleles were in very low frequency ranging from 0.011 to 0.106 (Table 1). Null alleles were present at more than one locus for each population except Mid-west Siberia (RAL and RNO), but there was no evidence of a large allele drop out (Table 1). Occurrence of null alleles at each locus showed generally low frequency less than 0.10 for most of populations. However, some loci showed various range of null alleles for certain populations as follows; 0.10 for the locus RT30 (SKM), IDVGA29 (SKJ) and BM757 (RYA), 0.30 for locus CSSM41 (SKJ, RPR and RUL), MB25 (SKM, RPR and MGN), Roe09 (SKM, RYA, and RUL), RT1 (SKM, RPR and RSO) and RT20 (SKJ, RPR and RYA). The highest frequency of null allele occurrence was found in the locus IDVGA8, with the null allele frequency of 0.60 for SKM, RPR, RSO, MGN, RKU, and RYA.

**Table 1:** Genetic characteristics of Siberian roe deer in each region/location across 12 microsatellite loci

	Region	N	MNA	*Ar*	*H*_E_	*H*_O_	*F*_IS_^a^	HWE *P* ^b^	Number of loci with null allele	NPA (Freq. rang)
East	**SKJ**	33	3.75	2.18	0.386	0.329	0.150*	0.000 (3)	3 (RT20, CSSM41, IDVGA29)	4 (0.016-0.106)
	**SKM**	31	6.58	3.48	0.596	0.451	0.247*	0.000 (7)	5 (RT1, RT30, Roe09, MB25, IDVGA8)	3 (0.016-0.065)
	**RPR**	30	7.42	3.67	0.623	0.490	0.217*	0.000 (7)	5 (RT1, RT20, MB25, CSSM41, IDVGA8)	4 (0.017-0.050)
	**RSMG**	21	7.00	5.67	0.598	0.500	0.169*	0.000 (4)	4 (RT1, MB25, BM757, IDVGA8)	7 (0.024-0.025)
	**RSO**	9	5.00	3.36	0.550	0.438	0.215*	0.000 (2)	2 (RT1, IDVGA8)	4 (0.056)
	**MGN**	12	5.67	3.66	0.628	0.544	0.138 ^NS^	0.000 (4)	2 (MB25, IDVGA8)	3 (0.042)
	**RYA**	18	5.33	3.26	0.553	0.459	0.175*	0.000 (4)	4 (RT20, Roe09, BM757, IDVGA8)	5 (0.031-0.094)
	**RARN**	12	3.92	3.87	0.560	0.503	0.107 ^NS^	0.000 (2)	1 (IDVGA8)	0
	**RAL**	5	2.92	2.81	0.541	0.471	0.144 ^NS^	0.003 (4)	- ^**c**^	-
	**RNO**	7	3.33	2.91	0.539	0.524	0.031 ^NS^	0.988 (0)	- ^**c**^	-
	**RURK**	44	4.92	3.73	0.534	0.495	0.075 ^NS^	0.000 (7)	3 (Roe09, CSSM41, IDVGA8)	3 (0.011-0.012)
	**RKU**	21	3.83	2.68	0.530	0.512	0.034 ^NS^	0.000 (6)	2 (Roe09, IDVGA8)	1 (0.025)
	**RUL**	23	4.42	2.82	0.522	0.478	0.085 ^NS^	0.000 (5)	2 (Roe09, CSSM41)	2 (0.022-0.024)
West	**Mean**	27	5.56	3.68	0.550	0.461	0.163	0.000 (5)	-	-

Number of individual per population (N), Allelic diversity (MNA, mean no. of alleles per locus), allelic richness (*Ar*), expected heterozygosity (*H*_E_) at Hardy-Weinberg equilibrium, observed heterozygosity (*H*_O_), inbreeding coefficient (*F*_IS_), and the probability (*P*) of being in Hardy-Weinberg equilibrium, null alleles, number of private alleles (NPA)

^a^ For *F*_IS_ within samples based on 2400 randomizations using the FSTAT program. NS: Not significant after adjusted nominal level (5 %) = 0.004

^b^ Probability values using the Fisher’s method implemented in the GENEPOP program. Number in parentheses indicates the no. of loci showing a significant departure (*P* <0.05) from Hardy-Weinberg equilibrium

^**c**^ Not determined due to small sample size

Measures of genetic diversity were generally high in Primorsky Krai, Russia (RPR) (mean no. of alleles per locus (MNA) = 7.42, Allelic richness (*Ar*) = 3.67, expected heterozygosity (*H*_E_) = 0.623) followed by Mainland Korea (SKM) and Northern Mongolia (MGN) (Table 1). The lowest genetic diversity was found in Jeju island, Korea (SKJ) (MNA = 3.75, *Ar* = 2.18, *H*_E_ = 0.386), followed by Mid-west Siberia (RAL and RNO) and West Siberia (RUL and RKU). Wilcoxon Signed Rank test revealed that allelic richness and expected heterozygosity were significantly higher in the East populations than in the West populations for the most population pairs (one tailed p < 0.05) (Additional file 1: Table S3, Figure S1).

All populations showed significant deviation of observed heterozygosity from heterozygosity expected under Hardy-Weinberg equilibrium in the direction of heterozygote deficiency except Novosibirsk, Russia (RNO) (Table 1). Inbreeding coefficient (*F*_IS_) estimates across all populations ranged from 0.031 to 0.247, and five populations (SKJ, SKM, RPR, RYA and RSO) were significantly deviated from zero (Table 1). Significant deviation in Hardy-Weinberg equilibrium (HWE) and *F*_IS_ could be due to the possibility of Whalund effect, inbreeding (due to non-random mating or subpopulations), and/or other anomaly such as the presence of null alleles.

### Genetic relationship and gene flow

ENA-corrected (excluding null alleles) and uncorrected pairwise *F*_ST_ are shown in Table 2, where these two estimates did not show significant differences (Wilcoxon Rank Sum Test; U = 987, *P* = 0.8401). Therefore, we used uncorrected pairwise *F*_ST_ for further analyses and interpretation of genetic differentiation of Siberian roe deer population. Pairwise *F*_ST_ values for 24 out of 44 population pairs are significantly different from 0 after corrections for multiple comparisons (*P* < 0.001) (Table 2). The lowest value of genetic differentiation was detected in SKM vs. MGN (*F*_ST_ = 0.025) and roe deer from Jeju Island, South Korea (SKJ), showed the highest degree of genetic differentiation to all others (mean pairwise *F*_ST_ = 0.349). When a comparison is made between two regions (West vs. Central and East), roe deer in Urals and Kurgan, Russia (RUL and RKU) showed relatively higher degrees of genetic differentiation with Mainland Korea (SKM), Primorsky Krai, Russia (RPR) and Central Siberia (RSO and MGN) (mean pairwise *F*_ST_ = 0.122). The effective number of migrants per generation (*N*_e_*m*) ranged from 0.4 (SKJ vs. RYA, RSO, RAL, RNO, RUL and RKU) to 103 (RPR vs. MGN) (Table 2). Roe deer in Jeju Island, Korea (SKJ) showed negligible levels of gene flow relative to all others.

**Table 2:** Pairwise *F*_ST_ and gene flow (*N*_e_ *m* in parentheses) estimates between geographic populations

	SKJ	SKM	RPR	RYA	RSO	MGN	RAL	RNO	RUL	RKU
**SKJ**	—	0.277 (0.7)	0.279 (0.7)	0.366 (0.4)	0.355 (0.5)	0.295 (0.6)	0.376 (0.4)	0.372 (0.4)	0.393 (0.4)	0.387 (0.4)
**SKM**	0.286*(0.6)	—	0.011 (23.1)	0.072 (3.3)	0.030 (8.2)	0.029 (8.3)	0.092 (2.5)	0.095 (2.4)	0.138 (1.6)	0.387 (2.0)
**RPR**	0.290*(0.6)	0.009^NS^(28.8)	—	0.046 (5.1)	0.007 (36.5)	0.011 (22.9)	0.065 (3.6)	0.081 (2.8)	0.115 (1.9)	0.095 (2.4)
**RYA**	0.373*(0.4)	0.068*(3.4)	0.044*(5.4)	—	0.038 (6.4)	0.056 (4.2)	0.054 (4.4)	0.045 (5.4)	0.054 (4.4)	0.055 (4.3)
**RSO**	0.366*(0.4)	0.020^NS^(12.1)	−0.005^NS^(inf)	0.041^NS^(5.8)	—	0.006 (42.4)	0.070 (3.3)	0.091 (2.5)	0.134 (1.6)	0.099 (2.3)
**MGN**	0.299*(0.6)	0.025*(10.0)	0.002 ^NS^(103)	0.051^NS^(4.6)	0.000^NS^(inf)	—	0.087 (2.6)	0.076 (3.0)	0.127 (1.7)	0.106 (2.1)
**RAL**	0.386*(0.4)	0.076^NS^(3.0)	0.055 ^NS^(4.3)	0.045^NS^(5.3)	0.058^NS^(4.1)	0.076^NS^(3.0)	—	0.065 (3.6)	0.107 (2.1)	0.116 (1.9)
**RNO**	0.380*(0.4)	0.088*(2.6)	0.070*(3.3)	0.039^NS^(6.2)	0.091^NS^(2.5)	0.070*(3.3)	0.057^NS^(4.2)	—	0.042 (5.8)	0.048 (5.0)
**RUL**	0.412*(0.4)	0.143*(1.5)	0.115*(1.9)	0.050*(4.8)	0.141*(1.5)	0.128*(1.7)	0.101^NS^(2.2)	0.035^NS^(7.0)	—	0.033 (7.4)
**RKU**	0.410*(0.4)	0.124*(1.8)	0.101*(2.2)	0.058*(4.1)	0.111*(2.0)	0.110*(2.0)	0.123^NS^(1.8)	0.045^NS^(5.3)	0.032^NS^(7.6)	—

*F*_ST_ estimates (Weir & Cockerham 1984) are below the diagonal and *F*_ST_ using the ENA correction are above the diagonal

Probability of being different than zero after corrections for multiple comparisons (*P < 0.001, NS: not significant)

**Fig. 2:**
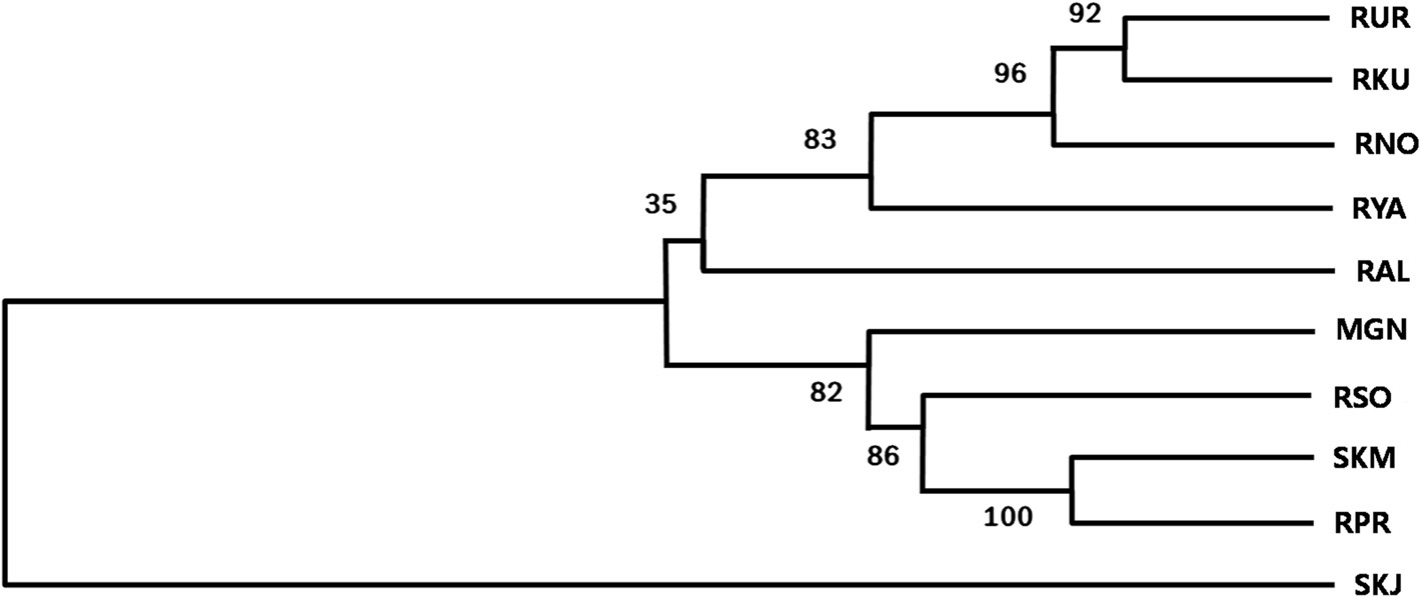
Relationship tree of Siberian roe deer from ten geographic locations. UPGMA tree was constructed based on Nei’s *D*_*A*_ genetic distance

**Fig. 3:**
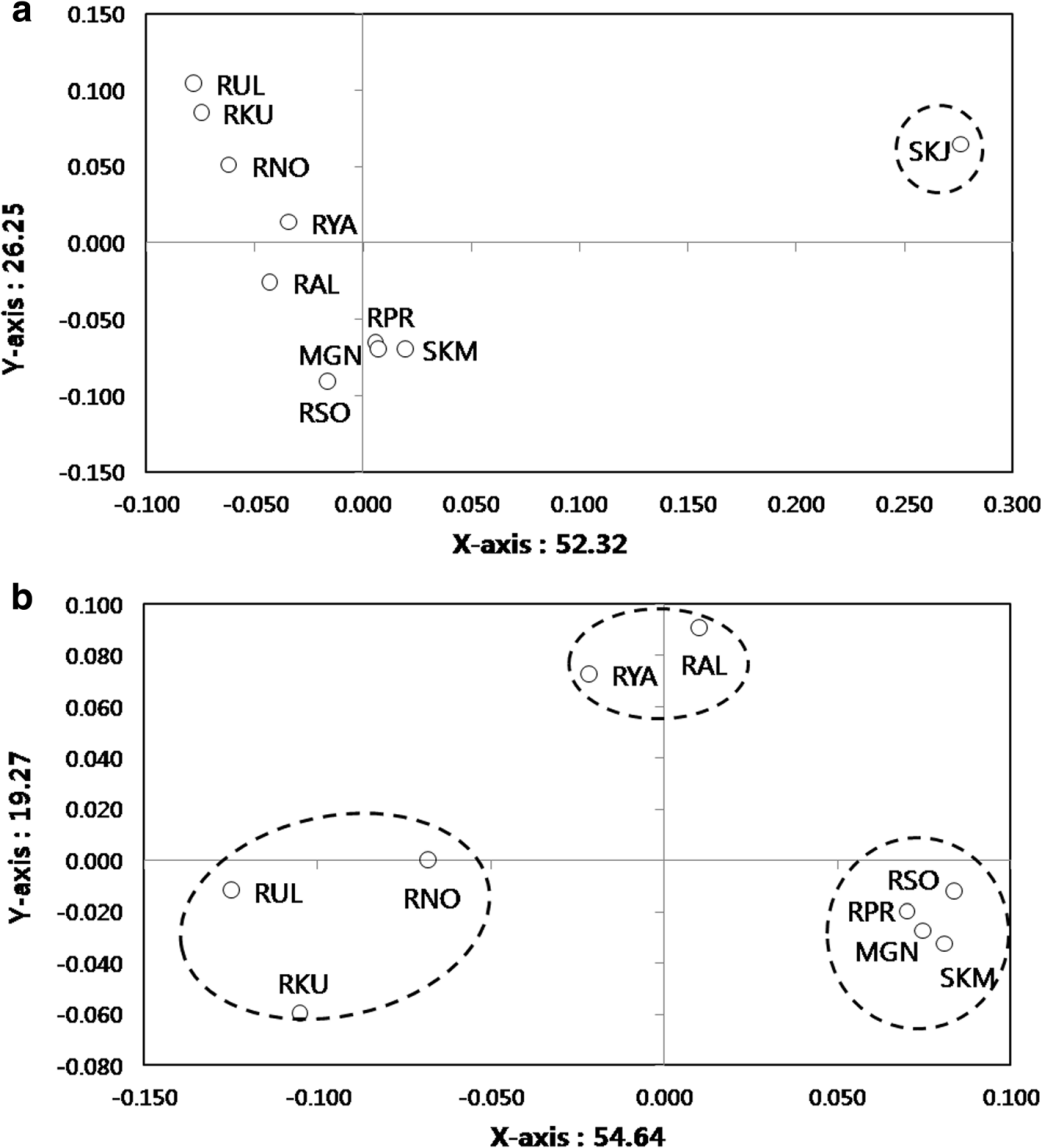
Scatter diagram of factor scores from a principal coordinate analysis of geographic locations. **a**: Analysis for all populations, **b**: Analysis after excluding roe deer from Jeju Island. The percentage of total variation attributed to each axis is indicated

UPGMA trees based on Nei’s *D*_*A*_ distances displayed topologies with three clusters (Fig. 2). Relationship tree displayed Mainland Korea, Eastern and Central Siberia populations (SKM, RPR, RSO and MGN) clustered together with high bootstrap support (82 %). However, the Jeju Island, South Korea (SKJ) population remains separated by long branches, possibly due to a founder effect. Principal coordinates analysis (PCA) for all populations supported the result from the relationship tree, revealing similar patterns among locations (Fig. 3a). PCA analysis performed without island population (SKJ) showed three clusters consisting of 1: Central and East (SKM, RPR, RSO and MGN), 2: West and Mid-west (RUL, RKU and RNO) and 3: Mid-west and Northeast (RAL and RYA) (Fig. 3b).

### Genetic structure

**Fig. 4:**
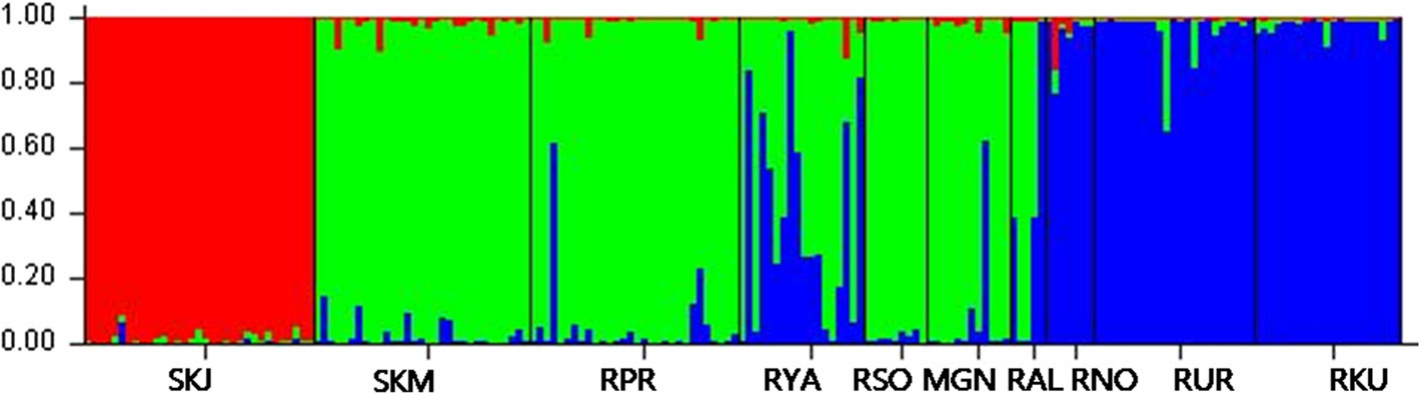
Bar plots for population structure estimates of Siberian roe deer. Population symbol on the x-axis indicates the putative population of sample origin. See Fig. 1 for location abbreviation. Each color denotes a cluster from STRUCTURE analysis

Bayesian model based clustering analysis identified three genetic clusters under the hierarchical island model suggested by the Evanno et al. [23] (Fig. 4). Initially, the highest ∆*K* was observed when *K* was set to 2, dividing into Jeju Island, South Korea (SKJ) and all other locations. When Jeju Island, South Korea (SKJ), was excluded to detect sub-structuring in remaining cluster, two additional genetic clusters were observed, which clearly discriminated the population in Central and Eastern Siberia (SKM, RPR, RSO and MGN) from those in the Urals region and West Siberia, Russia (RUL, RKU and RNO) populations. Mountain Altay, Russia (RAL) and Yakutia, Russia (RYA) displayed intermediate genetic composition between the Central/Eastern and Western population. Overall, structure analysis under the hierarchical island model revealed three genetic clusters consisting of 1: Jeju Island, South Korea (SKJ), 2: Central and East (SKM, RPR, RSO and MGN; Southeastern group), and 3: West and Mid-west (RUL, RKU and RNO; Northwestern group) with admixed genetic compositions between the clusters 2 and 3 for Mid-west (RAL) and Northeastern (RYA) population. A pie chart represented for each sampling location on the map, apart from roe deer from Jeju Island, South Korea (SKJ), displayed two different genetic compositions with an admixed population observed in border areas (Fig. 1).

Hierarchical analysis of molecular variance (AMOVA) analysis based on the geographical distance showed significant genetic differentiation (*F*_RT_ = 0.148) among regions, which was much higher than among population within regions (*F*_SR_ = 0.040) (Table 3A). Result based on the three clusters after two admixed regions (RYA and RAL) excluded presented greater difference in genetic differentiation among regions (*F*_RT_ = 0.200) (Table 3B), supporting the obvious genetic differentiation among three clusters; Jeju Island, Korea (SKJ), Eastern region (SKM, RPR, MGN and RSO) and Western region (RNO, RUL and RKU). In addition, AMOVA analysis based on the two clusters after Jeju and two admixed regions (RYA and RAL) excluded showed genetic differentiation among regions (*F*_RT_ = 0.093) and among population within regions (*F*_SR_ = 0.020) (Table 3C).

**Table 3:** Analysis of molecular variance (AMOVA) of the Siberian roe deer populations based on various geographic/genetic groupings (four geographic regions, three genetic clusters, and two geographic regions)

**A**								
**Source of variation**	**df**	**SS**	**MS**	**Est. Var.**	**%**	***F***-**Statistics**	**Value**	**P**-**Value**
**Among regions**	3	203.555	67.852	0.615	15	***F***_**RT**_	0.148	0.001
**Among pop**	6	50.962	8.494	0.142	3	***F***_**SR**_	0.040	0.001
**Among individuals**	179	733.874	4.100	0.710	17	***F***_**ST**_	0.182	0.001
**Within individuals**	189	506.500	2.680	2.680	65	***F***_**IS**_	0.209	0.001
**Total**	377	1494.892		4.147	100	***F***_**IT**_	0.354	0.001
**B**								
**Source of variation**	**df**	**SS**	**MS**	**Est. Var.**	**%**	***F***-**Statistics**	**Value**	**P**-**Value**
**Among regions**	2	192.296	96.148	0.853	20	***F***_**RT**_	0.200	0.001
**Among pop**	5	33.272	6.654	0.077	2	***F***_**SR**_	0.022	0.001
**Among individuals**	158	627.752	3.973	0.640	15	***F***_**ST**_	0.218	0.001
**Within individuals**	166	447.000	2.693	2.693	63	***F***_**IS**_	0.192	0.001
**Total**	331	1300.319		4.263	100	***F***_**IT**_	0.368	0.001
**C**								
**Source of variation**	**df**	**SS**	**MS**	**Est. Var.**	**%**	***F***-**Statistics**	**Value**	**P**-**Value**
**Among regions**	1	53.813	53.813	0.370	9	***F***_**RT**_	0.093	0.001
**Among pop**	5	33.272	6.654	0.071	2	***F***_**SR**_	0.020	0.001
**Among individuals**	126	524.919	4.166	0.645	16	***F***_**ST**_	0.111	0.001
**Within individuals**	133	382.500	2.876	2.876	73	***F***_**IS**_	0.183	0.001
**Total**	265	994.504		3.962	100	***F***_**IT**_	0.274	0.001

**A**: Four regions: Jeju Island (SKJ), East region (SKM, RPR), Central region (RYA, RSO, MGN) and West region (RAL, RNO, RUL, RKU). **B**: Three genetic clusters with two admixed populations (RYA and RAL) excluded: Jeju Island (SKJ), Eastern region (SKM, RPR, RSO, MGN) and Western region (RNO, RUL, RKU). **C**: Two geographic regions with SKJ and two admixed populations (RYA and RAL) excluded: Eastern region (SKM, RPR, RSO, MGN) and Western region (RNO, RUL, RKU)

df: degrees of freedom; SS: sum of squares; MS: mean squares; Est. Var.: estimated variance within and among populations

**Fig. 5:**
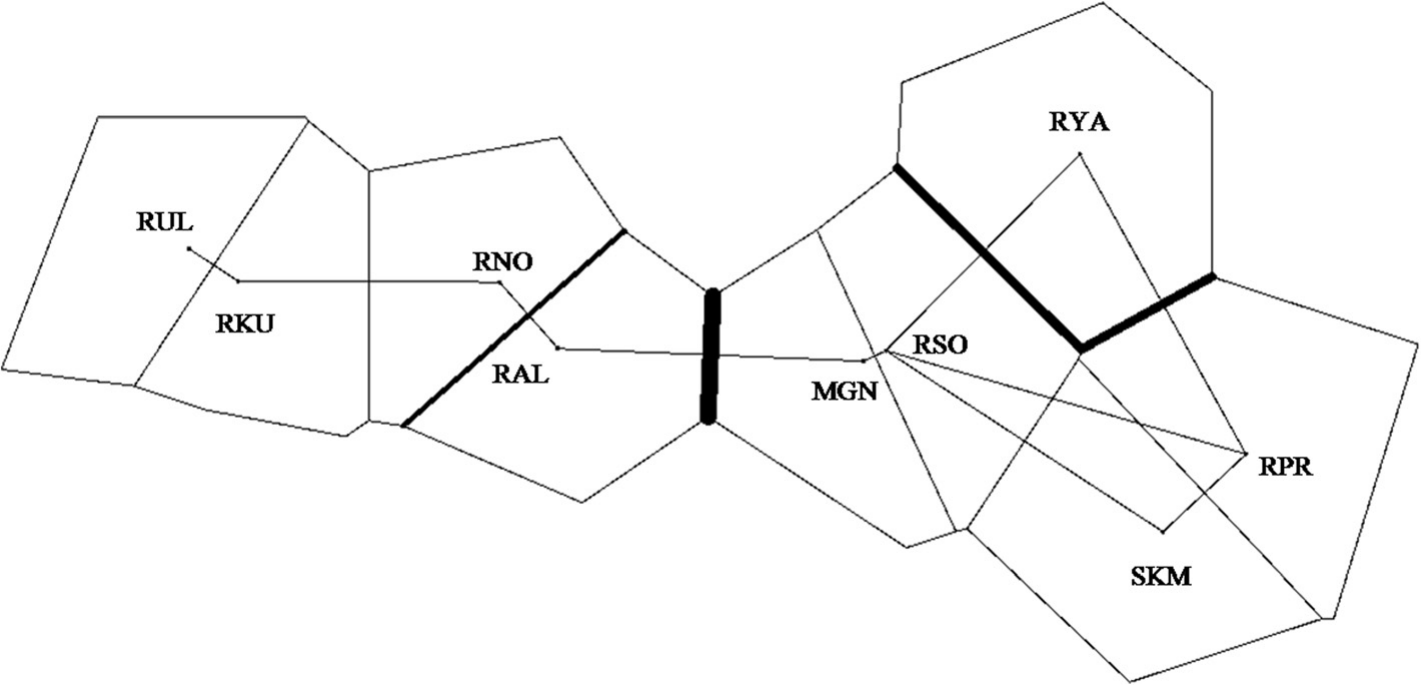
Areas of limited gene flow as estimated by BARRIER using Monmorier algorithm [70]. The genetic barriers are shown in bold lines, which are proportional to the intensity of the barriers

The Barrier analysis based on the pairwise *F*_ST_ verified three areas of relatively sharp change in genetic composition (Fig. 5). The first barrier separated the Eastern region (SKM, RPR, MGN and RSO) from West and Mid-west region (RAL, RNO, RUL and RKU) with supported by six to eleven loci. The second barrier separated Northeastern population (RYA) from all other populations with supported by three to eleven loci. The third barrier, supported by two to eleven loci, separated Mid-west population (RAL) from Western region (RNO, RUL and RKU).

**Fig. 6:**
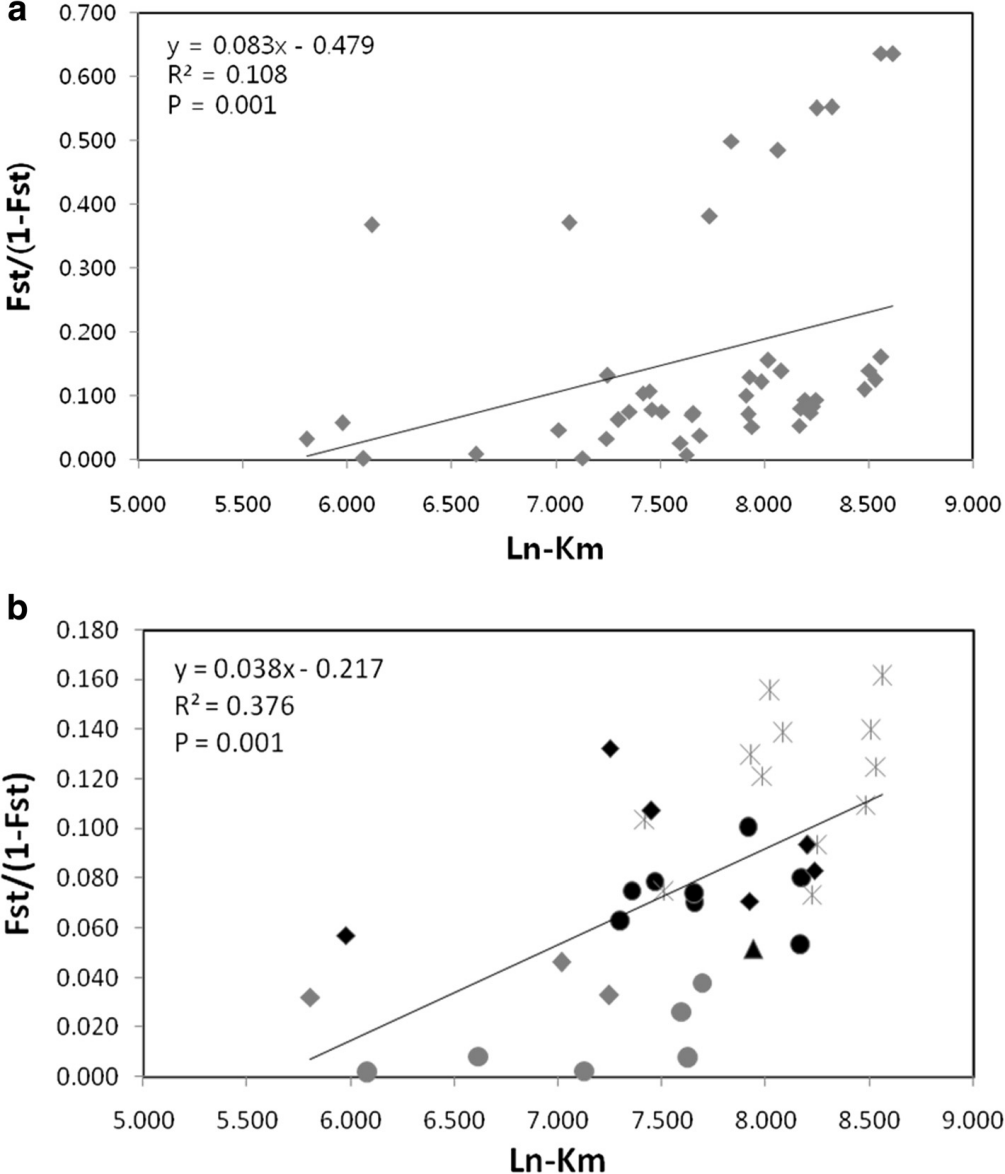
Regression of genetic distance on geographic distance between pairs of geographic Siberian roe deer populations. **a**: Analysis for all populations, **b**: Analysis after excluding roe deer from Jeju Island. Each diagram and color present pairs of population based on the structure result (two clusters). Mantel’s test for correlations was carried out with 999 permutations. Grey circle: within East cluster (SKM, RPR, MGN and RSO), Grey diamond: within West cluster (RNO, RUL and RKU), Black circle: between mixed populations (RAL and RYA) and East cluster, Black diamond: between mixed populations (RAL and RYA) and West cluster, Black triangle: within mixed populations (RAL and RYA), Asterisk: Between East and West cluster (opposite side of the mountains)

Regression of the genetic isolation by geographic distance (IBD) over all samples showed significant correlation in both with and without Jeju Island included (Fig. 6). However, relationship between genetic and geographic distances was increased as high as 3.5 fold when Jeju Island, Korea (SKJ), was removed, indicating that the distinct genetic differentiation of SKJ from other populations greatly decreased the IBD relationship. Also, IBD with marked pair of each population based on the two clusters (structure) showed slightly deviated point from standard linear which typically distributed on the low (pair of population within cluster) and high (pair of population between clusters) genetic distance (Fig. 6b).

To provide insights into the main causes of these three regions (SKJ, Eastern region and Western region) differentiation, statistical comparing p*R*_ST,_*F*_ST_ and *R*_ST_ values (drift vs mutation) were performed. p*R*_ST_ values were very similar to *F*_ST_ and permutation tests did not detect *R*_ST_ value significantly higher (p < 0.05) than p*R*_ST_ except one locus RT30 (Additional file 1: Table S4). This suggests that differentiation is caused mainly by drift. This result also ascertains the restricted level of gene flow between populations separated by the high mountain ridges and implies that *F*_ST_ should be a better estimator than *R*_ST_ of population differentiation for Siberian roe deer.

Three different measures of detecting population genetic bottlenecks revealed no evidence of a historical or recent bottleneck for nine populations (SKM, RPR, RYA, RSO, MGN, RAL, RNO, RUL and RKU) (Table 4). However, the event of a recent population bottleneck was detected in the Jeju Island, South Korea (SKJ) (Wilcoxon sign-rank test, two-phase mutation model (TPM) = 0.005), implying significant excess of heterozygosity relative to drift-mutation equilibrium. At the same time the Garza & Williamson’s [24] *M* values (0.765) and mode shift (none) tests did not show any evidence of genetic bottleneck. Bottleneck analysis suggested that all populations, except Jeju Island, South Korea (SKJ), were in the range of a historically stable population.

**Table 4:** Results of various tests to detect a recent population bottleneck event within geographic populations

Population	Wilcoxon sign-rank tests^a^	Mode shift	*M* ^b^
	TPM		
**SKJ**	0.005	None	0.765 (0.040)
**SKM**	0.266	None	0.885 (0.009)
**RPR**	0.519	None	0.929 (0.018)
**RYA**	0.380	None	0.777 (0.058)
**RSMG**	0.733	None	0.831 (0.037)
**RSO**	0.831	None	0.793 (0.052)
**MGN**	0.850	None	0.753 (0.048)
**RARN**	0.320	None	0.810 (0.057)
**RAL**	0.365	Shifted mode	0.769 (0.103)
**RNO**	0.206	Shifted mode	0.840 (0.055)
**RURK**	0.969	None	0.820 (0.058)
**RUL**	0.677	None	0.787 (0.073)
**RKU**	0.151	None	0.826 (0.069)

^a^One-tail probability for observed heterozygosity excess relative to the expected equilibrium heterozygosity (*H*_eq_), which is computed from the observed no. of alleles under drift-mutation equilibrium. TPM, two-phase model

^b^ *M* value and its variance (in parentheses) of Garza and Williamson. *M* = the mean ratio of the no. of alleles to the range of allele size

## Discussion

In this study, we investigated the variability of microsatellite loci to understand how different factors of genetic diversification such as isolation by distance, isolation by geographical barriers could affect the genetic diversity and population structure of Siberian roe deer in Northern Asia. Our study is based on samples from extensive geographic areas of Northern Asia, from Ural Mountains to the Korean Peninsula and Jeju Island, covering most of the species’ range to clarify the genetic relationships among populations from different geographical locations. Autosomal nuclear markers of microsatellites were employed to investigate the levels of genetic variation and genetic structuring of Siberian roe deer populations.

### Genetic diversity of Siberian roe deer

Relative comparison of genetic diversity estimates among other roe deer species/populations would be informative to understanding of the present genetic status of Siberian roe deer. Although different sets of microsatellite loci were employed, apart from populations in Jeju Island, South Korea (SKJ), most of Siberian roe deer populations revealed moderate levels of genetic diversity (*H*_E_ = 0.522 to 0.628), compared to those previously reported for European roe deer. Microsatellite diversity of European roe deer ranged from 0.17 to 0.79 in several locations from Italy, Britain and northern Germany (*H*_E_ = 0.17 to 0.58 [11], *H*_E_ = 0.59 to 0.62 [18], and *H*_E_ = 0.74 to 0.79 [25], respectively). However, because the different sets of microsatellites were employed in diversity estimates and this may cause an inherent ascertainment bias that can vary among primer pairs, especially in different species, it should be interpreted with caution.

During the 20th century, many of the local Siberian roe deer populations were significantly abated as a result of human interference [26-30]. However, present data on the genetic diversity of Siberian roe deer suggests that the historical population reduction was transient, and its effects on the genetic diversity of the populations were insignificant. Result of bottleneck test also supported the lack of evidence for bottleneck event, except in the Jeju Island population (See below), indicating general stability of Siberian roe deer populations in continental Asia.

Different measures of microsatellite variability are consistently high in populations from East and Central Asia compared to West Siberia (Table 1). One reasonable assumption is that areas to the south and east of Siberia have function as refugia for roe deer during glacial periods. Several vertebrate species were also reported to have high levels of mitochondrial DNA variations in eastern Russia compared with those of surrounding areas [31]. Combination of cold open steppes with forested areas in south and east of Siberia may have resulted in highly diverse faunas [32], which could provide preservation and diversification of genetic lineages. However, phylogeographic and archaeological inference with additional samples from different geographical regions, using various marker systems, such as mtDNA and nuclear genes, should be implemented to precisely determine the role of this region as refugia.

Roe deer from Jeju Island, South Korea (SKJ) showed the lowest level of genetic diversity among Siberian roe deer that were sampled in this study. This presumably is due to the geographic isolation and historical population fluctuations on Jeju Island. Roe deer inhabited in Jeju Island during the last glacial maximum (LGM) when there was a bridge between the island and the Korean peninsula. It is probable that a relatively small group of animals was founded in the island after the last glacial periods, which led to reduced genetic diversity due to processes such as founder effect and genetic drift. Human interference, such as excessive hunting and poaching, could be another possible cause of the genetic deprivation in Jeju population. The roe deer population in Jeju gradually declined to near extinction in the early 1970s because of continuous hunting and poaching [33]. Since the 1980s, Jeju Special Self-Governing Province and Jeju citizens has been active in conservation for roe deer such as providing food during winter, removing traps, and clamping down on poaching [34, 35]. Consequently, the roe deer population in Jeju increased to 5,000 individuals in 1992 and climbed to 12,881 individuals in 2009 [33]. The effect of recent fluctuations of roe deer population in Jeju Island on its genetic diversity is supported by the Bottleneck tests (Table 4). Therefore, continuous monitoring of genetic diversity would be essential for effective management and conservation of Siberian roe deer in Jeju Island.

### Genetic structure and gene flow

Present studies of genetic structure and differentiation among Siberian roe deer populations clearly display the existence of genetically distinct three clusters which comprise of the southeastern group (SKM, RPR, RSO and MGN), northwestern group (RUL, RKU and RNO) and Jeju Island population in Korea (SKJ). Such pattern of genetic structure is well in accordance with distribution of the two subspecies, *C. p. pygargus* and *C. p. tianschanicus*, suggested by previous study [36]. Recently, mitochondrial DNA sequence and nuclear IRBP (Interphotoreceptor retinoid binding protein) data has been presented that Jeju Island population to another subspecies, *C. p. ochracea* [37]. The genetic makeups of the two populations (RYA and RAL) are indicative of admixture of the two groups (southeastern and northwestern groups); however, a small sample size limits ultimate defining of their genetic status.

A previous study [2] proposed three major factors that may limit the geographical distribution of Siberian roe deer. The first factor is geographical barriers consisting of major mountain ridges (Altai, Sayans and Stanovoye) and the Lake Baikal (Fig. 1), which also delineate geographical ranges of two subspecies (*C. p. pygargus* and *C. p. tianschanicus*). The second factor is the depth of snow and duration of the snowy period [2, 38, 39] and last factor is the predominant vegetation type of the region, such as taiga, tundra, and desert [2]. These three factors and their interaction presumably limited further spread of roe deer, but probably first factor is the most important for the formation of genetic groups or subspecies. The other possible reason of it is that the mountain ridges could serve as refugia during periods of climate change (e. g. during the glacial maximums). In the periods of climatic optimums different genetic lineages could spread from the mountains in different areas resulting in formation of genetically different groups, possibly subspecies. However, this assumption need to additional phylogenetic studies will be required.

Barrier analysis that detected change genetic composition was also support limited gene flow in the major mountain ridges (Fig. 5). Southeastern group (SKM, RPR, RSO and MGN) and Northwestern group (RUL, RKU and RNO) supported relatively high frequency and fallowed by genetically admixed two populations (RYA and RAL) in the border areas. Besides, results of the Isolation by distance (IBD) (Fig. 6b) displayed that about 38 % of the genetic variation is explained by geographical distances between locations over the entire continent of Asia, which fits the hierarchical island model, suggesting modern genetic structure resulted from natural processes [2, 10, 40, 41]. Additionally, different pattern of distribution in the IBD scatter plot between and within groups (southeastern and northwestern groups) ascertains the effect of mountains ridges on the restricted level of gene flow between groups. Thus, mountain ridges of the southern Siberia have limited gene flow between Southeastern (SKM, RPR, RSO and MGN) and Northwestern (RUL, RKU and RNO) groups, leading to current genetic structure.

It should be noted that the Altay population (RAL) is located in the border area of two subspecies and shows the admixed pattern of two genetic clusters. This population is genetically related to both groups (Southeastern and Northwestern) and likely has historical and ongoing gene flow with adjacent locations (Fig. 1). A previous study of mitochondrial DNA [42] proposed that roe deer in Altai Mountain might experience multiple population replacements, stressing the role of the Altai Mountain as a physical boundary separating *C. p. pygargus* and *C. p. tianschaniscus*. This speculation is based on the genetic heterogeneity of Siberian roe deer in the Altai Mountains, and relatively stable climatic conditions of the region compared to other Siberian regions during the Pleistocene [42]. However, to resolve the question of border area, additional population genetic studies with more samples from areas at a finer geographic scale will be required.

Roe deer population in Yakutia, Russia (RYA), were established as a result of natural radiation from the southern parts of geographical range and could originate from both *C. p. pygargus* and *C. p. tianschaniscus* [43]. This assumption complies with the genetic structure of the Yakutian population obtained in this study and is also confirmed by the previous studies using morphology and karyotype [44, 45].

Roe deer from Jeju Island, South Korea (SKJ) are genetically divergent from all other Siberian roe deer, including those on the Korean mainland. The Jeju Island population was isolated from the mainland population since LGM, and as a result, there has been no gene flow between these two locations. Thus, the present genetic feature of the Jeju Island population was derived as a consequence of long-term geographical isolation and adaptation to island environment. Cases where Jeju island populations showing unique genetic and/or morphological features was also described for other mammal species such as wild boar (*Sus scrofa*), striped field mouse (*Apodemus agrarius chejuensis*) and Siberian weasel (*Mustela sibirica*) [46]. Future studies of this isolated population would contribute to understanding the effect of peripheral isolation on microevolution in Cervidae.

Our results do not coincide with the recent phylogeographic findings [19] that demonstrated no apparent geographical structuring for Siberian roe deer sampled from vast geographic areas of Eurasia. Variability of mtDNA control region suggested that the Siberian roe deer in Asia has undergone genetic admixture and appears to show no apparent geographic barriers to gene flow [19]. This difference could be due to the sensitivity of molecular markers and disparate interpretation owing to insufficient sample size and different modes of inheritance. The microsatellites are highly polymorphic and autosomal nuclear markers with biparental inheritance, and are more appropriate to delineate genetic structure of recently diverged populations.

### Management and conservation Implications

Overall, this study suggests that at least three distinct management units may exist for the Siberian roe deer populations in Asia [47]: Northwest genetic group (RUL, RKU and RNO, partially corresponding to *C. p. pygargus* subspecies), southeast genetic group (SKM, RPR, RSO and MGN, corresponding to *C. p. tianschanicus*) and Jeju Island genetic group. Future planning of management and/or conservation policies, including ex situ population breeding, translocation and reintroduction programs, need to consider the distinctiveness of the three genetic groups in the Siberian roe deer species. Strict application of management unit concept for the two admixed populations (RYA and RAL) might be relaxed, or postponed until more detailed studies focusing on these populations are performed.

The roe deer population in Jeju Island, Korea (SKJ) needs special attention due to its low level of genetic diversity compared to those of continental populations. The Jeju Island population seems to be thriving at the present time, despite the low level of heterozygosity. The current size of the Jeju roe deer population is estimated to be around 12,881 [33] and considered to be over-populated in the island. However, considering the deprived level of genetic diversity, it is probable that the Jeju population might be vulnerable to epidemic diseases or any change of environment in the future. Therefore, it is recommended that both the genetic and physical health statuses of the population are closely monitored. Artificial translocation of roe deer individuals from the mainland Korea to Jeju Island to increase genetic diversity of Jeju population is not recommended because these two populations are genetically highly differentiated and should be regarded as separate management units.

Herbivorous animals such as roe deer play an important role in the ecosystem, providing a prey for large carnivores. Therefore, proper genetic management of Siberian roe deer populations and continuous monitoring of its genetic status is critical for maintaining healthy ecosystem. It is important to stress that systematic cooperation between countries where Siberian roe deer inhabit (Russia, Kazakhstan, Mongolia, China, North Korea and South Korea) is imperative for effective maintenance of genetic diversity and gene flow of Siberian roe deer. In particular, cooperative management of border area is important not only for the roe deer itself but also for a number of endangered large carnivore species.

For example, Siberian roe deer is one of the main prey animal of Amur leopard (*Panthera pardus orientalis*) in the border area among Russia, China and North Korea [48, 49]. Thus maintaining healthy roe deer population in this transboundary region is crucial for the survival of Amur leopard, which is one of the most severely endangered subspecies of large Felidae species in the world [49–53]. The status of the Siberian roe deer population in North Korea remains unknown and the gene flow has been discontinued along the Demilitarized Zone (DMZ) of North and South Korean border for more than five decades. This situation would have negative impacts on the long-term persistence of the Siberian roe deer in Korean peninsula and the restoration efforts of Amur leopard and tiger populations in this region. Siberian roe deer also serve as an important prey species for other carnivores like Amur tigers, gray wolves, lynxes, dholes, bears, as well as foxes, martens, eagles and wild boars [51, 54]. Thus, proper management of roe deer populations in northern Asian continent will also benefit many other species, and eventually, the biodiversity of the entire region.

## Conclusion

In conclusion, the present study reveals that Siberian roe deer inhabiting Asia is composed of genetically distinct populations (Southeast, Northwest and Jeju Island, Korea) and East–west gradient in genetic diversity. As a whole, geographical barriers, as well as the genetic isolation as a function of geographic distance ascertain restricted level of gene flow among roe deer populations over the whole continent of Asia. Two genetically admixed populations, however, also reside in the border areas between the two genetically distinct groups. Knowledge on the present status of genetic structure and genetic diversity of Siberian roe deer has important implications on the ecological and geographical impact on genetic characteristics of Siberian roe deer. The insights obtained from this study can be applied in management and conservation of local populations of Siberian roe deer in Asia and raise the necessity of continuous monitoring of genetic status of such important animals.

## Methods

### Sample collection and DNA extraction

A total of 189 individuals of *C. pygargus* were collected from ten locations in Russia, Mongolia and South Korea (Fig. 1). Jeju, South Korea (SKJ), Mainland South Korea (SKM), Primorsky Krai, Russia (RPR), Yakutia, Russia (RYA), surroundings of Sokhondinsky Zapovednik (nature reservation), Russia (RSO), Northern part of Mongolia (MGN), Altaisky Krai, Russia (RAL), Novosibirskaya Oblast’, Russia (RNO), Sverdlovskaya Oblast’, Ural, Russia (RUL) and Kurganskaya Oblast’, Russia (RKU). This experimental work was conducted with permission by the Conservation Genome Resource Bank for Korean Wildlife (CGRB) that provided the roe deer samples for this study. All samples were legally collected and deposited into CGRB. The procedures involving animal samples followed the guidelines by Seoul National University Institutional Animal Care and Use Committee (SNU IACUC). Tissue (muscle, skin and liver) and blood samples were collected across the current distribution range of *C. pygargus* from 2001 to 2011, and were frozen at −70 °C deep freezer in the CGRB or stored in ethanol until DNA extraction. Genomic DNA was extracted from individual sample using the DNeasy tissue and blood kit (Qiagen, Valencia, CA) following the manufacturer’s protocol.

### Microsatellite analysis

A total of 12 microsatellite loci were used and tested for genotyping and genetic analysis of *C. pygargus* sampled. Microsatellite markers previously developed from rein deer (RT1, RT20, RT23, RT24, RT30), cattle (MB25, BM757, CSSM41, IDNGA8, IDNGA29), and European roe deer (Roe01, Roe09) have proved to be polymorphic in Siberian roe deer, and were used through the cross-species amplification in this study (Additional file 1: Table S2). Genomic DNA was amplified for genotyping under the following conditions. The touchdown profile for the PCR amplification was at 94 °C for 15 min, followed by 20 cycles at 94 °C for 30 S, 65 °C for 60 S, and 72 °C for 30 S, with annealing temperature decreased by 0.5 °C per cycle to 55 °C. The touchdown cycles were followed by an additional 25 cycles at 94 °C for 30 S, 55 °C for 1 min, 72 °C for 30 S, and a final extension at 72 °C for 20 min. The PCR reaction mixture contained MgCl_2_ (2 mM), dNTP (each 0.2 mM), and i-Star *Taq* DNA polymerase (0.025 U) of iNtRON biotechnology Inc (Korea). One of three (Hex, 6-Fam, Tamra) fluorescently-labeled M13 primers (0.26 pmol), unlabeled M13-tailed forward primer (0.13 pmol), and reverse primer (0.26 pmol) were also added to the reaction tubes. All amplifications were implemented in a volume of 15 μl in TaKaRa thermal cyclers. Alleles were determined by ABI Prism3730 XL DNA Analyzer (Applied Biosystemsinc, USA) using GENESCAN-500 [Rox] size standard and analyzed GeneMapper version 3.7 (Applied Biosystemsinc, USA).

### Data analysis

#### Summary statistics

Ten locations were used for basic analyses to obtain the summary statistics, and to improve statistical power for certain analysis like Bottleneck test, six locations with geographical proximity and small sample size were further pooled into three locations such as, (RSMG: RSO & MGN), (RARN: RAL & RNO) and (RURK: RUL & RKU). The number of all alleles per locus and population (MNA), observed heterozygosity (*H*_O_) and expected heterozygosity (*H*_E_) in Hardy-Weinberg equilibrium were estimated for each locus using the Microsatellite Toolkit, version 3.0 [55]. Allelic richness (*Ar*), *F*-statistics (*F*_IS,_*F*_ST_) [56] and genotype linkage disequilibrium for all pair of loci in population were determined using the program FSTAT, version 2.9.3 [57]. Allelic Richness is one of important measures of genetic diversity and is calculated based on a minimum sample size of each population to compensate for the differences in sample size among populations. Wilcoxon signed rank test was employed to assess differences in allelic richness and expected heterozygosity that are corrected by small sample sizes using the STATISTIX version 8.1 (Analytical Software, Statistix; Tallahassee, FL, USA, 2000). The number of loci with null alleles was assessed using MICRO-CHECKER [58]. Occurrence of null alleles can lead to diminution in genetic diversity and inflate genetic differentiation among population [59]. Null alleles can be common owing to ascertainment bias and sequence variation especially when microsatellites from cross-species amplification are used. The number of private alleles and genetic characteristics of 12 microsatellite loci for ten regional samples were determined using the GenAlEx version 6.1 [60]. The program CERVUS, version 2.0 was used to calculate the polymorphism information content (PIC), observed heterozygosity (*H*_O_) and expected heterozygosity (*H*_E_) of each locus [61]. Deviations from Hardy-Weinberg equilibrium (HWE) for each geographic population were evaluated using the exact probability test [62] using the Fisher procedure calculated by GENEPOP, version 3.3 [63].

#### Gene flow measures

The pattern of gene flow between populations was measured using two different approaches. First, the effective number of migrants per generation (*N*_e_*m*) between populations was calculated from with the following formula: *N*_e_*m* = (1 − *F*_ST_) / 4*F*_ST_ [64], where *N*_e_ is the effective population size and *m* is the migration rate. This gene flow (*N*_e_*m*) estimate is an approximation of a particular theoretical model (Island model) at equilibrium that migration occurs at the same rate with equal population size. *F*_ST_ is a measure of genetic differentiation between populations and allows estimation of relatively long-term gene flow based on allele frequency distributions. Pairwise *F*_ST_ between populations and their significance calculated using the program FSTAT version 2.9.3 [57]. Also, pairwise *F*_ST_ were corrected by the ENA method (excluding null alleles) using the FREENA software [65]. The difference between the ENA corrected and uncorrected *F*_ST_ values was evaluated by the Wilcoxon rank sum test using the STATISTIX version 8.1 (Analytical Software, Statistix; Tallahassee, FL, USA, 2000).

#### Genetic relationship

The genetic relationship between populations was evaluated by the Nei’s genetic distances (*D*_A_) [66] based on allele frequencies using the program DISPAN [67]. Genetic relationship trees were constructed by unweighted pair group method with the arithmetic mean (UPGMA) [68] based on *D*_A_ distance with 1000 bootstrap replications to test the validity of tree topologies. Principal coordinate analysis (PCA) was conducted using the covariance matrix of allele frequencies using the GENALEX version 6.1 [60]. Two principal values with the first and second highest factor scores were employed to construct a scatter diagram to visualize genetic relationships among populations. The GENALEX version 6.1 was further used to carry out hierarchical analysis of molecular variance (AMOVA) of genetic differentiation among populations and regions, and *F*-statistics (*F*_RT_, *F*_SR_, *F*_ST_, *F*_IS_ and *F*_IT_). According to the geographical distance, ten roe deer populations were divided into four main regions for the AMOVA analysis: Jeju Island, South Korea (SKJ), East region (SKM, RPR), Central region (RYA, RSO, MGN) and West region (RAL, RNO, RUL and RKU). Besides, according to the structure result (three clusters), eight roe deer populations were divided into three main regions excluding the two admixed populations (RYA, RAL) for the AMOVA analysis: Jeju Island, South Korea (SKJ), Eastern region (SKM, RPR, RSO and MGN) and Western region (RNO, RUL and RKU). Additionally, seven populations were divided into two main regions with SKJ and two admixed populations (RYA and RAL) excluded: Eastern region (SKM, RPR, RSO, MGN) and Western region (RNO, RUL, RKU). Significance level was calculated by the permutation procedure (999 permutations).

#### Population structure

Existence of population genetic structuring was evaluated using the model-based Bayesian clustering method in the program STRUCTURE version 2.3.4 [69], which infers the number of genetic clusters (*K*) without prior information about population origin. This method calculates independent assessments of each individual for each cluster. The log-likelihood data [Ln Pr (*X/K*)] was estimated for given *K* between 1 and 10 with ten independent runs set by 1,000,000 Markov chain Monte Carlo (MCMC) iterations followed by burn-in period of 100,000 iterations. The “real” value of *K* within the dataset was estimated from the Ln Pr (*X/K*) according to the method of Evanno et al. [23], in which log-likelihood values and variance from each replicate of *K* were used to calculate ∆*K*. An ad hoc statistic test in this parameter was used in simulations to identify the true number of genetic clusters, which offers accurate means to selecting *K* instead of choosing a *K* with the highest log probability that could lead to overestimated *K* [23]. Existence of Isolation-by-distance (IBD) [64] was obtained by the regression of genetic distance (*F*_ST_ / (1-*F*_ST_)) on geographic distance (Ln-Km) between pairs of populations. The correlations for two variables and probability were carried out using the Mantel’s test in GENALEX version 6.1 and significance was determined based on 999 permutations [60].

We also applied Monmonier’s maximum difference algorithm to highlight geographical features with obvious genetic discontinuity between populations using the program BARRIER version 2.2 [70]. The data from nine populations except Jeju island, Korea (SKJ) were analyzed to detect putative barriers of gene flow among the populations. Geographical coordinates were used for each population and connected by Delauney triangulation using a pairwise *F*_ST_ genetic matrix. We conducted the analysis using *F*_ST_ for each of the eleven microsatellite loci; exclude IDVGA29 due to low polymorphism, to make sure that the barriers were not verified with strong differentiation at only few loci. Each locus indicates how many support a given barrier and putative genetic boundaries were identified across the geographical landscapes. Pairwise *F*_ST_, *R*_ST_ and p*R*_ST_ (*R*_ST_ computed after allele size permutation test with 1000 randomizations) were calculated per each population and locus to estimate the main causes of population differentiation in Siberian roe deer using program SPAGeDi [71, 72]. *R*_ST_ was compared against the distribution of p*R*_ST_ values.

#### Bottleneck detection

Three different approaches were used to detect molecular evidence of historical population bottleneck. First, we tested for deviations of expected heterozygosity (*H*e) relative to heterozygosity expected at drift-mutation equilibrium (*H*_eq_) by Wilcoxon sign-rank tests (∝ = 0.05, ∝ = 0.01) [73] using the BOTTLENECK version 1.2.02 [74, 75]. During bottlenecks, the number of rare alleles is reduced faster than the heterozygosity at polymorphic loci due to drift [66]. Thus the bottleneck test can detect this disparity when *H*_e_ becomes larger than *H*_eq_, because *H*_eq_ reflects allele number and sample size. We used a two-phase mutation model (TPM) [76] using a setting of 10 % multiple-step mutations and 90 % single-step mutations with 1,000 iterations. Secondly, we checked out a mode-shift in distributions of allele frequencies from the l-shaped distribution under the mutation-drift equilibrium, expecting distorted distribution under the recent population bottleneck [77].

Lastly, *M* value of Garza & Williamson’s [24] was calculated for each population to detect the long-term decrease of population size using the program AGARST version 3.3 [78]. *M* is the mean ratio of the total number of alleles to the range of allele size. This test is useful for detecting a bottleneck further in the past (>100 generations). Meta-analysis for natural populations revealed that historically reduced or founded populations had *M*-ratio < 0.68, but stable populations showed *M* > 0.82.

## Additional file

Additional file 1:
**Table S1.** Genetic characteristics of 12 microsatellite loci for Siberian roe deer from seven geographic regions in Asia. See Fig. 4 for sampling regions. **Table S2:** Source information and characteristics of 12 microsatellite markers obtained from cross-species amplification. **Table S3:** Wilcoxon signed rank test to assess differences in allelic richness (*Ar*) and expected heterozygosity that are corrected by small sample sizes (UH_E_) (one-tailed p-value). **Figure S1:** Bar graph of allelic diversity (*Ar*) and expected heterozygosity that are corrected by small sample sizes (UH_E_) in eight Siberian roe deer population. **Table S4:** Differentiation among three regions (cluster) of Siberian roe deer estimated by pairwise *R*_ST_, mean p*R*_ST_ and *F*_ST_ values per locus and multilocus.

## Abbreviations

SKJ: Jeju South Korea
SKM: Mainland South Korea
RPR: Primorsky Krai Russia
RYA: Yakutia Russia
RSO: Sokhondinsky Zapovednik Russia
MGN: Northern part of Mongolia
RAL: Altaisky Krai Russia
RNO: Novosibirskaya Oblast’ Russia
RUL: Sverdlovskaya Oblast’ Ural, Russia
RKU: Kurganskaya Oblast’ Russia
PIC: Polymorphism information content
AMOVA: Analysis of molecular variance
PCA: Principal coordinates analysis
HWE: Hardy-Weinberg equilibrium
MNA: Mean number of alleles per locus
ENA: Excluding null alleles
IBD: Isolation by geographic distance
TPM: Two-phase mutation model
