# Supplementary material for: Genetic diversity and genetic structure of the Siberian roe deer (Capreolus pygargus) populations from Asia
Source: BMC Genet. 2015 Aug 18;16:100. doi: 10.1186/s12863-015-0244-6 (PMC4539716; doi:10.1186/s12863-015-0244-6)
Supplement: Additional file 1: — Table S1. Genetic characteristics of 12 microsatellite loci for Siberian roe deer from seven geographic regions in Asia. See Fig. 4 for sampling regions. Table S2: Source information and characteristics of 12 microsatellite markers obtained from cross-species amplification. Table S3: Wilcoxon signed rank test to assess differences in allelic richness (Ar) and expected heterozygosity that are corrected by small sample sizes (UHE) (one-tailed p-value). Figure S1: Bar graph of allelic diversity (Ar) and expected heterozygosity that are corrected by small sample sizes (UHE) in eight Siberian roe deer population. Table S4: Differentiation among three regions (cluster) of Siberian roe deer estimated by pairwise R ST, mean pR ST and F ST values per locus and multilocus. [file 12863_2015_244_MOESM1_ESM.doc]

**SUPPORTING INFORMATION**

**Genetic diversity and genetic structure of the Siberian roe deer (*Capreolus pygargus*) populations from Asia**

Yun Sun Lee, Nickolay Markov, Inna Voloshina, Alexander Argunov, Damdingiin Bayarlkhagva, Jang Geun Oh, Yong-Su Park, Mi-Sook Min, Hang Lee^*^ and Kyung Seok Kim^*^

Table S1. Genetic characteristics of 12 microsatellite loci for Siberian roe deer from seven geographic regions in Asia. See figure 1 for sampling regions.

| **Locus** | **SKJ** | **SKM** | **RPR** | **RYA** | **RSMG** | **RARN** | **RURK** | **Mean** |
| --- | --- | --- | --- | --- | --- | --- | --- | --- |
| **RT1** |  |  |  |  |  |  |  |  |
| No. of alleles | 1 | 13 | 13 | 9 | 15 | 6 | 9 | 9.429 |
| *H*_O_ | 0.000 | 0.742 | 0.607 | 0.882 | 0.667 | 0.750 | 0.698 | 0.621 |
| *H*_E_ | 0.000 | 0.871 | 0.893 | 0.815 | 0.907 | 0.785 | 0.800 | 0.724 |
| HWE *P*-value | NA | 0.0106 | 0.0000 | 0.4639 | 0.0000 | 0.2227 | 0.0010 | - |
| **RT20** |  |  |  |  |  |  |  |  |
| No. of alleles | 4 | 4 | 5 | 5 | 4 | 4 | 5 | 4.429 |
| *H*_O_ | 0.107 | 0.484 | 0.483 | 0.556 | 0.667 | 0.667 | 0.690 | 0.522 |
| *H*_E_ | 0.427 | 0.648 | 0.729 | 0.739 | 0.676 | 0.740 | 0.642 | 0.657 |
| HWE *P*-value | 0.0000 | 0.1252 | 0.0027 | 0.0463 | 0.6315 | 0.3087 | 0.2435 | - |
| **RT23** |  |  |  |  |  |  |  |  |
| No. of alleles | 4 | 4 | 5 | 1 | 4 | 3 | 3 | 3.429 |
| *H*_O_ | 0.545 | 0.419 | 0.517 | 0.000 | 0.524 | 0.250 | 0.477 | 0.390 |
| *H*_E_ | 0.606 | 0.563 | 0.467 | 0.000 | 0.422 | 0.226 | 0.428 | 0.388 |
| HWE *P*-value | 0.2140 | 0.0056 | 0.8054 | NA | 1.0000 | 1.0000 | 0.6717 | - |
| **RT24** |  |  |  |  |  |  |  |  |
| No. of alleles | 3 | 5 | 5 | 5 | 4 | 4 | 5 | 4.429 |
| *H*_O_ | 0.303 | 0.667 | 0.600 | 0.788 | 0.571 | 0.750 | 0.773 | 0.635 |
| *H*_E_ | 0.326 | 0.669 | 0.721 | 0.719 | 0.670 | 0.736 | 0.655 | 0.642 |
| HWE *P*-value | 0.4697 | 0.4372 | 0.2165 | 0.3950 | 0.2238 | 0.8595 | 0.2946 | - |
| **RT30** |  |  |  |  |  |  |  |  |
| No. of alleles | 4 | 15 | 18 | 10 | 12 | 4 | 8 | 10.143 |
| *H*_O_ | 0.758 | 0.645 | 0.833 | 0.765 | 0.900 | 0.500 | 0.705 | 0.729 |
| *H*_E_ | 0.549 | 0.898 | 0.911 | 0.858 | 0.823 | 0.663 | 0.773 | 0.782 |
| HWE *P*-value | 0.0551 | 0.0000 | 0.0305 | 0.3283 | 1.0000 | 0.1733 | 0.0012 | - |
| **Roe01** |  |  |  |  |  |  |  |  |
| No. of alleles | 3 | 3 | 3 | 3 | 3 | 2 | 2 | 2.714 |
| *H*_O_ | 0.333 | 0.516 | 0.433 | 0.706 | 0.381 | 0.833 | 0.886 | 0.584 |
| *H*_E_ | 0.282 | 0.398 | 0.346 | 0.503 | 0.316 | 0.500 | 0.500 | 0.407 |
| HWE *P*-value | 1.000 | 0.2582 | 0.4458 | 0.2104 | 1.0000 | 0.0764 | 0.0000 | - |
| **Roe09** |  |  |  |  |  |  |  |  |
| No. of alleles | 2 | 2 | 2 | 2 | 4 | 2 | 2 | 2.286 |
| *H*_O_ | 0.531 | 0.194 | 0.633 | 0.188 | 0.619 | 0.364 | 0.182 | 0.387 |
| *H*_E_ | 0.500 | 0.458 | 0.499 | 0.482 | 0.517 | 0.397 | 0.499 | 0.479 |
| HWE *P*-value | 1.0000 | 0.0016 | 0.2705 | 0.0303 | 0.8623 | 1.0000 | 0.0000 | - |
| **MB25** |  |  |  |  |  |  |  |  |
| No. of alleles | 2 | 2 | 2 | 2 | 2 | 2 | 2 | 2.000 |
| *H*_O_ | 0.030 | 0.161 | 0.233 | 0.333 | 0.190 | 0.500 | 0.205 | 0.236 |
| *H*_E_ | 0.030 | 0.398 | 0.499 | 0.346 | 0.499 | 0.444 | 0.283 | 0.357 |
| HWE *P*-value | NA | 0.0018 | 0.0037 | 1.0000 | 0.0068 | 1.0000 | 0.0857 | - |
| **BM757** |  |  |  |  |  |  |  |  |
| No. of alleles | 7 | 15 | 18 | 15 | 17 | 10 | 12 | 13.429 |
| *H*_O_ | 0.576 | 0.839 | 0.867 | 0.625 | 0.810 | 0.833 | 0.841 | 0.770 |
| *H*_E_ | 0.641 | 0.908 | 0.920 | 0.906 | 0.922 | 0.875 | 0.828 | 0.857 |
| HWE *P*-value | 0.1089 | 0.0000 | 0.0073 | 0.0000 | 0.0529 | 0.0000 | 0.0063 | - |
| **CSSM41** |  |  |  |  |  |  |  |  |
| No. of alleles | 7 | 3 | 3 | 4 | 4 | 2 | 2 | 3.571 |
| *H*_O_ | 0.281 | 0.290 | 0.200 | 0.364 | 0.286 | 0.167 | 0.114 | 0.243 |
| *H*_E_ | 0.437 | 0.350 | 0.413 | 0.318 | 0.255 | 0.375 | 0.312 | 0.351 |
| HWE *P*-value | 0.0000 | 0.2186 | 0.0017 | 1.0000 | 1.0000 | 0.0899 | 0.0002 | - |
| **IDVGA8** |  |  |  |  |  |  |  |  |
| No. of alleles | 5 | 13 | 12 | 7 | 13 | 7 | 5 | 8.714 |
| *H*_O_ | 0.485 | 0.452 | 0.400 | 0.308 | 0.381 | 0.417 | 0.295 | 0.391 |
| *H*_E_ | 0.624 | 0.878 | 0.885 | 0.737 | 0.906 | 0.698 | 0.548 | 0.753 |
| HWE *P*-value | 0.1088 | 0.0000 | 0.0000 | 0.0017 | 0.0000 | 0.0004 | 0.0000 | - |
| **IDVGA29** |  |  |  |  |  |  |  |  |
| No. of alleles | 3 | 1 | 3 | 1 | 2 | 1 | 4 | 2.143 |
| *H*_O_ | 0.000 | 0.000 | 0.069 | 0.000 | 0.000 | 0.000 | 0.071 | 0.020 |
| *H*_E_ | 0.140 | 0.000 | 0.067 | 0.000 | 0.091 | 0.000 | 0.070 | 0.053 |
| HWE *P*-value | 0.0004 | NA | 1.0000 | NA | 0.0244 | NA | 1.0000 | - |

Table S2. Source information and characteristics of 12 microsatellite markers obtained from cross-species amplification.

| **Locus** | **Size range** | **NA** | ***H*_E_** | ***H*_O_** | **PIC** | **Origin** | **GenBank**  **Accession no.** | **Reference** |
| --- | --- | --- | --- | --- | --- | --- | --- | --- |
| **RT1** | 221-259 | 18 | 0.890 | 0.587 | 0.877 | Rein deer (*Rangifer tarandus*) | U90737 | Wilson *et al*., (1997). |
| **RT20** | 235-245 | 6 | 0.767 | 0.509 | 0.724 | Rein deer (*Rangifer tarandus*) | U90744 | Wilson *et al*., (1997). |
| **RT23** | 200-208 | 5 | 0.494 | 0.433 | 0.459 | Rein deer (*Rangifer tarandus*) | U90745 | Wilson *et al*., (1997). |
| **RT24** | 210-220 | 6 | 0.766 | 0.622 | 0.722 | Rein deer (*Rangifer tarandus*) | U90746 | Wilson *et al*., (1997). |
| **RT30** | 201-249 | 22 | 0.914 | 0.738 | 0.905 | Rein deer (*Rangifer tarandus*) | U90749 | Wilson *et al*., (1997). |
| **Roe01** | 152-160 | 5 | 0.438 | 0.580 | 0.359 | Roe deer (*Capreolus capreolus*) | AF164070 | Fickel & Reinsch (2000). |
| **Roe09** | 195-201 | 4 | 0.503 | 0.378 | 0.381 | Roe deer (*Capreolus capreolus*) | AF166358 | Fickel & Reinsch (2000). |
| **MB25** | 219-221 | 2 | 0.501 | 0.201 | 0.375 | Cattle (*Bos taurus*) |  | Kappes *et al*., (1997). |
| **BM757** | 170-220 | 24 | 0.933 | 0.775 | 0.926 | Cattle (*Bos taurus*) | G18473 | Kappes *et al*., (1997). |
| **CSSM41** | 124-150 | 10 | 0.502 | 0.227 | 0.461 | Cattle (*Bos taurus*) | U03816 | Slate *et al*., (1998). |
| **IDVGA8** | 229-259 | 16 | 0.887 | 0.391 | 0.875 | Cattle (*Bos taurus*) | Z27074 | Slate *et al*., (1998). |
| **IDVGA29** | 155-163 | 4 | 0.063 | 0.029 | 0.062 | Cattle (*Bos taurus*) | X85048 | Slate *et al*., (1998). |

Number of alleles (NA) and PIC were obtained from analysis for 189 Siberian roe deer.

Table S3. Wilcoxon signed rank test to assess differences in allelic richness (*Ar*) and expected heterozygosity that are corrected by small sample sizes (UH_E_) (one-tailed p-value).

|  | **SKJ** | **SKM** | **RPR** | **RYA** | **RSO** | **MGN** | **RAL** | **RNO** | **RUL** | **RKU** |
| --- | --- | --- | --- | --- | --- | --- | --- | --- | --- | --- |
| **SKJ** |  | 0.013 | 0.013 | 0.055 | 0.066 | 0.008 | 0.066 | 0.105 | 0.077 | 0.039 |
| **SKM** | 0.011 |  | 0.046 | 0.416 | 0.190 | 0.066 | 0.276 | 0.326 | 0.121 | 0.032 |
| **RPR** | 0.012 | 0.013 |  | 0.046 | 0.013 | 0.451 | 0.077 | 0.077 | 0.013 | 0.013 |
| **RYA** | 0.032 | 0.378 | 0.105 |  | 0.535 | 0.121 | 0.535 | 0.535 | 0.158 | 0.055 |
| **RSO** | 0.055 | 0.276 | 0.105 | 0.496 |  | 0.003 | 0.416 | 0.496 | 0.382 | 0.525 |
| **MGN** | 0.005 | 0.046 | 0.416 | 0.205 | 0.032 |  | 0.091 | 0.077 | 0.008 | 0.011 |
| **RAL** | 0.046 | 0.051 | 0.032 | 0.051 | 0.166 | 0.013 |  | 0.215 | 0.489 | 0.451 |
| **RNO** | 0.066 | 0.088 | 0.032 | 0.123 | 0.166 | 0.013 | 0.254 |  | 0.382 | 0.382 |
| **RUL** | 0.091 | 0.046 | 0.002 | 0.046 | 0.205 | 0.000 | 0.416 | 0.416 |  | 0.489 |
| **RKU** | 0.105 | 0.003 | 0.001 | 0.021 | 0.256 | 0.000 | 0.158 | 0.121 | 0.256 |  |

Below diagonal: *p*-value in *Ar*, Above diagonal: *p*-value in UHe


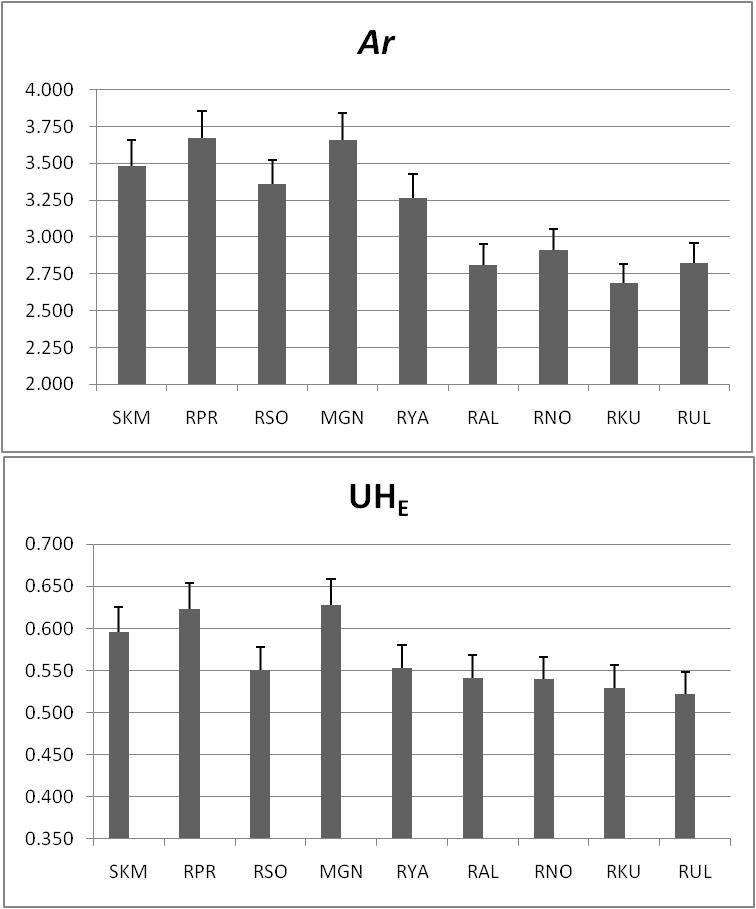


Figure S1. Bar graph of allelic diversity (*Ar*) and expected heterozygosity that are corrected by small sample sizes (UH_E_) in eight Siberian roe deer population

Table S4. Differentiation among three regions (cluster) of Siberian roe deer estimated by pairwise *R*_ST_, mean p*R*_ST_ and *F*_ST_ values per locus and multilocus.

| **Locus** | **NA** | ***R*_ST_** | **p*R*_ST_ (C.I.)** ^b^ | ***F*_ST_** |
| --- | --- | --- | --- | --- |
| Multilocus | 10.17 | 0.171 NS ^a^ | 0.119 (0.055-0.196) | 0.124 |
| **RT1** | 18 | 0.177 NS | 0.128 (0.007-0.246) | 0.167 |
| **RT20** | 6 | 0.111 NS | 0.086 (-0.012-0.162) | 0.101 |
| **RT23** | 5 | 0.107 NS | 0.081 (0.030-0.133) | 0.094 |
| **RT24** | 6 | 0.101 NS | 0.107 (0.016-0.186) | 0.123 |
| **RT30** | 22 | 0.308* | 0.140 (0.038-0.276) | 0.168 |
| **Roe01** | 5 | 0.048 NS | 0.074 (0.032-0.090) | 0.079 |
| **Roe09** | 4 | 0.009 NS | 0.031 (0.008-0.048) | 0.033 |
| **MB25** | 2 | 0.201 NS | 0.201 (0.201-0.201) | 0.201 |
| **BM757** | 24 | -0.012 NS | 0.053 (-0.018-0.160) | 0.063 |
| **CSSM41** | 10 | 0.021 NS | 0.107 (0.019-0.183) | 0.128 |
| **IDVGA8** | 16 | 0.068 NS | 0.117 (-0.007-0.305) | 0.140 |
| **IDVGA29** | 4 | -0.016 NS | -0.023 (-0.034--0.013) | -0.021 |

^a^ probability values of allele size permutation tests on *R*_ST_ (**P* < 0.01, NS: not significant).

^b^ 95% confidence interval (C.I.) is obtained after 1000 random permutation of the allele size.

NA: Number of alleles
